# Zoonotic Threat of G4 Genotype Eurasian Avian-Like Swine Influenza A(H1N1) Viruses, China, 2020

**DOI:** 10.3201/eid2808.212530

**Published:** 2022-08

**Authors:** Min Gu, Kaibiao Chen, Zhichuang Ge, Jun Jiao, Tianyu Cai, Suhan Liu, Xiaoquan Wang, Xinan Jiao, Daxin Peng, Xiufan Liu

**Affiliations:** Yangzhou University, Yangzhou, China (M. Gu, K. Chen, Z. Ge, J. Jiao, T. Cai, S. Liu, X. Wang, X. Jiao, D. Peng, X. Liu);; Jiangsu Co-innovation Center for Prevention and Control of Important Animal Infectious Diseases and Zoonoses, Yangzhou (M. Gu, X. Wang, X. Jiao, D. Peng, X. Liu)

**Keywords:** influenza, H1N1, swine influenza, viruses, G4 genotype, Eurasian avian-like H1N1, zoonoses, China

## Abstract

We investigated genetic and biologic characteristics of 2 Eurasian avian-like H1N1 swine influenza viruses from pigs in China that belong to the predominant G4 genotype. One swine isolate exhibited strikingly great homology to contemporaneous human Eurasian avian-like H1N1 isolates, preferential binding to the human-type receptor, and vigorous replication in mice without adaptation.

Pigs have long been considered a crucial genetic mixing vessel for influenza A viruses (IAVs) of different hosts (*1*) because of the dual expression of human (SAα-2,6Gal) and avian (SAα-2,3Gal) viral receptors on their respiratory epithelium. Swine IAVs such as H1N1 and H3N2 subtypes sporadically infect humans and are prone to cause bidirectional interspecies transmission at the swine–human interface (*2*–*5*). So far, Eurasian avian-like (EA) H1N1 has dominated prevalence in pig herds in China and caused >10 human infections (*6*–*9*). In particular, the dominant genotype 4 (G4) EA H1N1 containing 2009 pandemic influenza A(H1N1) polymerase basic (PB) 1 and 2, polymerase acid (PA), nucleoprotein (NP), and matrix (M) genes, plus the triple-reassortant (TR) nonstructural (NS) gene, is thought to be a candidate virus of potential pandemic (*10*,*11*). Indeed, a case of human infection with G4 EA H1N1 was reported in Yunan Province, China, in 2021 (*8*). It is imperative to conduct surveillance on swine IAVs and evaluate their risk to public health.

## The Study

During monthly surveillance of swine IAVs in China during October–December 2020, we collected a total of 376 nasal swab samples from apparently healthy pigs in a slaughterhouse accommodating swine from neighboring regions (Jiangsu, Shandong, and Anhui Provinces in eastern China). We detected H1 subtype swine influenza virus in 9 of those by real-time reverse transcription quantitative PCR; 2 were confirmed as hemagglutinin (HA) positive after inoculating into MDCK cells (*12*). We further evaluated these 2 swine IAV isolates, A/swine/Jiangsu/HD11/2020 (H1N1) [HD11] and A/swine/Anhui/HD21/2020 (H1N1) [HD21], for their genetic and biologic characteristics.

The genome sequences of HD11 and HD21 deposited in the GenBank database (accession no. OL744678–93) shared 95.4%–99.0% nucleotide identities across the coding regions of 8 genes. We performed searches of those sequences on BLAST (https://blast.ncbi.nlm.nih.gov/Blast.cgi) and the GISAID database (http://platform.gisaid.org) to present a more comprehensive scene of the homologous reference influenza viruses. As shown by the closest BLAST hits (Table 1), HD11 and HD21 were not only highly related to swine origin IAVs collected during 2012–2018 but also remarkably similar to contemporaneous human H1N1 isolates from 2020 and 2021.

**Table 1:** Comparison of 2 G4 Eurasian avian-like H1N1 swine isolates from pigs in China with similar influenza viruses retrieved from the GISAID and GenBank databases*

Gene and isolate	Most homologous sequence in GISAID				Most homologous sequence in GenBank		
	Virus strain	% Similarity			Virus strain	% Similarity	
		nt	aa			nt	aa
PB2							
HD11	A/Sichuan/01208/2021(H1N1)	99.25	99.47		A/swine/Liaoning/PJ89/2014(H1N1)	97.72	98.69
HD21	A/Sichuan/01208/2021(H1N1)	97.11	98.03		A/swine/Liaoning/PJ89/2014(H1N1)	97.54	98.29
PB1							
HD11	A/Shandong/00204/2021(H1N1)	99.60	100.00		A/swine/Liaoning/CY102/2014(H1N1)	97.98	98.42
HD21	A/Hubei-Wujiagang/1324/2020(H1N1)	97.58	98.68		A/swine/Liaoning/CY102/2014(H1N1)	97.71	98.68
PA							
HD11	A/Tianjin/00030/2020(H1N1)	99.67	99.86		A/swine/Liaoning/PJ43/2014(H1N1)	97.44	99.30
HD21	A/swine/China/Qingdao/2018(H1N1)	97.49	99.02		A/swine/China/Qingdao/2018(H1N1)	97.49	99.02
HA							
HD11	A/Tianjin/00030/2020(H1N1)	99.47	99.47		A/swine/Liaoning/CY102/2014(H1N1)	97.47	97.18
HD21	A/Tianjin/00030/2020(H1N1)	98.71	98.59		A/swine/Liaoning/CY102/2014(H1N1)	97.30	97.35
NP							
HD11	A/Tianjin/00030/2020(H1N1)	99.73	100.00		A/swine/Guangxi/NS1402/2012(H3N2)	97.80	98.20
HD21	A/Tianjin/00030/2020(H1N1)	98.00	98.60		A/swine/Guangdong/NS2883/2012(H3N2)	97.80	99.00
NA							
HD11	A/Shandong/00204/2021(H1N1)	99.57	99.36		A/swine/Ningjin/03/2014(H1N1)	97.02	95.96
HD21	A/Sichuan/01208/2021(H1N1)	97.02	97.45		A/swine/Liaoning/PJ43/2014(H1N1)	96.67	95.74
M							
HD11	A/Tianjin/00030/2020(H1N1)	99.69	99.70		A/swine/Shandong/LY142/2017(H1N1)	98.78	98.78
	A/Sichuan/01208/2021(H1N1)						
HD21	A/Tianjin/00030/2020(H1N1)	98.47	98.48		A/swine/Shandong/LY142/2017(H1N1)	98.57	99.39
	A/Sichuan/01208/2021(H1N1)	98.47	98.48				
NS							
HD11	A/Shandong/00204/2021(H1N1)	100.00	100.00		A/swine/Guangxi/1874/2012(H3N2)	97.97	96.43
	A/Sichuan/01208/2021(H1N1)	100.00	100.00				
HD21	A/Hubei-Wujiagang/1324/2020(H1N1)	97.49	95.00		A/swine/China/Qingdao/2018(H1N1)	97.14	95.00

*HD11 is the isolate A/swine/Jiangsu/HD11/2020(H1N1); HD21 is the isolate A/swine/Anhui/HD21/2020(H1N1). GISAID, https://www.gisaid.org. HA, hemagglutinin; M, matrix; NA, neuraminidase; NP, nucleoprotein; NS, nonstructural protein; PA, polymerase acid; PB, polymerase basic.

We constructed a phylogenetic gene tree analysis with H1N1 reference strains to confirm the intimate genetic relationship between these 2 swine IAVs and human viruses (Appendix Figure 1). In each tree, HD11 consistently clustered with 3 human H1N1 viruses, A/Tianjin/00030/2020(H1N1), A/Shandong/00204/2021(H1N1), and A/Sichuan/01208/2021(H1N1). As for HD21, the virus aggregated closely with the HD11-involved subbranch in PB2, HA, NP, NA, and M gene trees but gathered more intimately with another 3 human H1N1 viruses containing A/Hubei-Wujiagang/1324/2020(H1N1), A/Gansu-Xifeng/1143/2021(H1N1) and A/Gansu-Xifeng/1194/2021(H1N1) in PB1, PA, and NS gene trees. Taken together, HD11 and HD21 were both closest to contemporaneous human H1N1 strains, and they uniformly possessed the EA H1N1-like HA and NA genes, pandemice influenza–like RNP (PB2, PB1, PA, and NP) and M genes, and TR-like NS gene that made the G4 type gene constellation. We observed that 2 additional swine reference viruses of A/swine/Shandong/LY142/2017(H1N1) and A/swine/China/Qingdao/2018(H1N1) assembled tightly with the HD11/HD21 cluster, further supporting the possibility of IAV interspecies transmission from swine to human.

**Figure 1:**
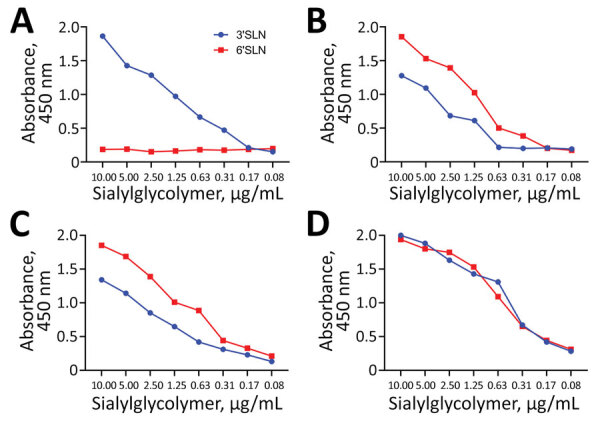
Receptor-binding property of 2 G4 Eurasian avian-like influenza A(H1N1) swine isolates from pigs in China. A) The control virus A/mallard/Huadong/S/2005(H5N1) (HDS05) showed an absolute preference for avian-type SAα-2,3Gal. B) The control virus A/Jiangsu/202/2010(H3N2) (JS202) displayed double affinities to both human-type SAα-2,6Gal and avian-type SAα-2,3Gal, but with an overt bias toward SAα-2,6Gal. C) The tested virus A/swine/Jiangsu/HD11/2020(H1N1) (HD11) resembled the human-origin JS202 to possess an obviously advantageous avidity for SAα-2,6Gal over SAα-2,3Gal. D) The tested virus A/swine/Anhui/HD21/2020(H1N1) (HD21) exhibited comparable binding capacity to SAα-2,6Gal and SAα-2,3Gal without apparent preference. The solid-phase direct binding ELISA assay with the synthetic sialyl glycopolymers containing either 3′SLN-PAA and 6′SLN-PAA was applied to estimate the virus binding to avian-type SAα-2,3Gal and human-type SAα-2,6Gal, respectively. The data shown are representative of 3 independent binding experiments. SLN, sialyl-N-acetyllactosamine.

The 2 G4 genotype EA H1N1 swine isolates both propagated well in specific-pathogen-free chicken embryos with virus titers per 0.1mL >9 log_10_ 50% egg infectious dose (EID_50_) (Table 2). However, HD11 replicated much better than HD21 in MDCK cells through the titration of the 50% culture infectious dose (TCID_50_) value and virus growth at 12-hours intervals across 12–60 hours postinfection (hpi). At >24 hpi, HD11 had generated more than 5 log_10_ TCID_50_ and reached a peak of 7 log_10_ TCID_50_ at 36 hpi, whereas the titer of HD21 virus remained at the relatively lower level <5 log_10_ TCID_50_ until the endpoint.

**Table 2:** Virus replication of 2 G4 Eurasian avian-like H1N1 swine isolates from pigs in China in vitro and in vivo*

Virus strain	log_10_ EID_50_/0.1 mL	log_10_ TCID_50_/0.1 mL	Virus growth in MDCK cells, mean titer ±SD, log_10_ TCID_50_/0.1 mL†						Virus replication in infected mice, mean titer +SD, log_10_ copies/μL‡						
									3 dpi				5 dpi		
			12 hpi	24 hpi	36 hpi	48 hpi	60 hpi		Lung	Turb	Brain		Lung	Turb	Brain
HD11	9.5	7.5	3.872§ +0.645	5.041 +0.219	7.000¶ +0.441	5.556# +0.096	5.667** +0.000		5.679# +0.355	4.295** +0.181	2.495 +0.318		3.828 +1.484	2.385 +0.219	2.703 +0.661
HD21	9.375	5.769	3.055 +0.481	4.389 +0.096	4.556 +0.096	4.556 +0.096	4.444 +0.096		3.894 +0.195	2.008 +0.988	1.667 +0.537		4.550 +0.53	2.334 +0.221	2.692 +0.132

*We conducted 2-way analysis of variance in Prism software version 8 (GraphPad, https://www.graphpad.com) for virus titer comparison between HD11 and HD21 groups in each time point in cells or each tissue of the same sampling day in mice. dpi, days postinfection; EID_50_, 50% egg infectious dose; HD11, A/swine/Jiangsu/HD11/2020(H1N1); HD21, A/swine/Anhui/HD21/2020(H1N1); hpi, hours postinfection; TCID_50_, 50% tissue culture infectious dose (determined in MDCK cells); turb, turbinate. †MDCK monolayers were infected with HD11 and HD21 at a multiplicity of infection (MOI) of 0.1. The virus titers of cell supernatants collected at different time points of 12, 24, 36, 48, and 60 h postinfection were determined via the TCID_50_ assay in MDCK cells. ‡Three 6-week-old BALB/c mice per group challenged with 10^6.0^ EID_50_ virus in 50 μL volume were euthanized to collect tissue samples including the lung, turbinate, and brain for virus titration on 3 and 5 d postinoculation. The viral load expressed with virus copies in tissue homogenates was measured through the real-time quantitative reverse transcription PCR method as described (*12*). §p<0.05. ¶p<0.0001. #p<0.01. **p<0.001.

Subsequently, we conducted a solid-phase direct binding ELISA assay with the synthetic glycopolymer-based receptor mimics Neu5Aca2-3Galb1-4GlcNAcb(3ʹSLN)-PAA-biotin and Neu5Aca2-3Galb1-4GlcNAcb(6ʹSLN)-PAA-biotin (GlycoTech, https://www.glycotech.com) to evaluate the viral receptor-binding preference as previously described (*13*). We used 1 avian H5N1 virus and 1 human seasonal H3N2 virus as controls; the avian virus displayed a complete 3ʹsialyl-N-acetyllactosamine (SLN) affinity, whereas the human virus possessed a dual binding property to both 3ʹSLN and the more advantageous 6ʹSLN (Figure 1). Unlike HD21, which was endowed with comparable avidity between 3ʹSLN and 6ʹSLN, HD11 resembled the binding feature of the human-origin H3N2 virus that preferentially binds the human-type SAα-2,6Gal (Figure 1).

**Figure 2:**
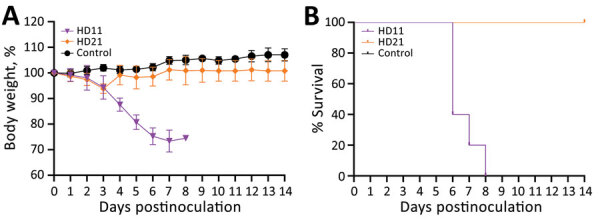
Pathogenicity of 2 G4 Eurasian avian-like influenza A(H1N1) swine isolates from pigs in China in BALB/c mice. A) Body weight change of infected mice. B) Survival curve of infected mice. Two groups of five 6-week-old BALB/c mice were inoculated intranasally with A/swine/Jiangsu/HD11/2020(H1N1) (HD11) or A/swine/Anhui/HD21/2020(H1N1) (HD21) at a dose of 10^6^ 50% egg infectious dose/50 µL. Another 5 mice mock-infected with phosphate-buffered saline were served as control. Body weight change and survival rate were recorded daily until 14 days postinoculation, and mice that lost ≥25% of the initial body weight were humanely euthanized.

We then investigated the pathogenicity of HD11 and HD21 in mice. We infected 6-week-old BALB/c mice in groups of 5 intranasally with 10^6.0^ EID_50_ virus dose or mock-inoculated them with phosphate-buffered saline (PBS). We monitored body weight changes and clinical symptoms of the mice daily for 14 days. We humanely euthanized an additional 3 challenged mice per group and analzyed them for virus load in tissues at 3 and 5 days postinfection (dpi). Mice in the control group displayed a steady increase in body weight, the HD21 group experienced a slightly transient weight loss on 3 dpi, and all mice survived during the entire experiment (Figure 2). In contrast, HD11 resulted in a steady decrease in body weights starting at 1 dpi, and all died within 8 days. In addition, we observed that both HD11 and HD21 replicated efficiently in the lungs without prior adaptation and readily disseminated into nasal turbinates and the brain (Table 2). Of note, the virus load in respiratory tissues of HD11-infected mice was significantly higher (p<0.01 in lungs and p<0.001 in turbinates) than that of HD21-infected mice on 3 dpi. On 5 dpi, we observed no significant difference in virus titers in the 3 tissues of the mice infected with these 2 isolates. Moreover, HD11 infections increased the mRNA levels of inflammatory cytokines, including interleukin 6 and 10, interferon β and γ, MX1, and C-X-C motif chemokine ligand 10 11 on 3 dpi, 5 dpi, or both, more dramatically than HD21 virus. Both HD21 and HD11 infections increased tumor necrosis factor α expression at relatively low levels (Appendix Figure 2).

## Conclusions

Homology alignment and phylogenetic tree construction analysis suggest that HD11 and HD21, two G4 EA H1N1 swine IAVs isolated in 2020 in China, are strongly related to recent human-origin EA H1N1 viruses. In particular, HD11 had higher affinity for human-type 6ʹSLN at the level that is equivalent to the human seasonal H3N2 virus. Moreover, HD11 replicated much faster in vitro in MDCK cells and in vivo in the lung than di HD21 and was highly pathogenic to BALB/c mice, as evidenced by its lethality, higher viral loads in pulmonary tissues, and higher levels of inflammatory cytokines in the lung. We propose that the HD11-like G4 swine isolates whose genomic sequences share great homology with that of contemporaneous human EA H1N1 viruses may lead to interspecies transmission. Therefore, the public health threat from the zoonotic G4 EA H1N1 viruses should not be underestimated.

Appendix Additional information about the potential zoonotic threat of G4 genotype Eurasian avian-like swine influenza A(H1N1) viruses, China, 2020.
