# Supplementary material for: Zoonotic Threat of G4 Genotype Eurasian Avian-Like Swine Influenza A(H1N1) Viruses, China, 2020
Source: Emerg Infect Dis. 2022 Aug;28(8):1664–8. doi: 10.3201/eid2808.212530 (PMC9328894; doi:10.3201/eid2808.212530)
Supplement: Appendix — Additional information about the potential zoonotic threat of G4 genotype Eurasian avian-like swine influenza A(H1N1) viruses, China, 2020. [file 21-2530-Techapp-s1.pdf]

Article DOI: https://doi.org/10.3201/eid2808.212530

Zoonotic Threat of G4 Genotype Eurasian Avian-Like Swine Influenza A(H1N1) Viruses, China, 2020

Appendix

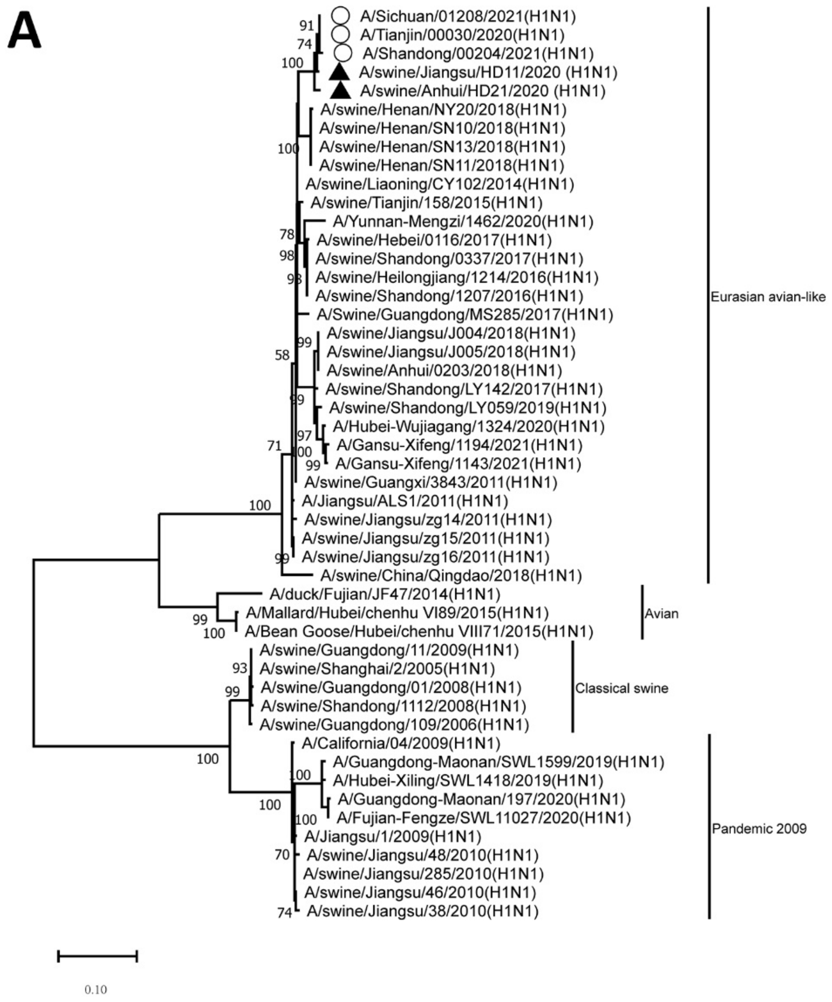

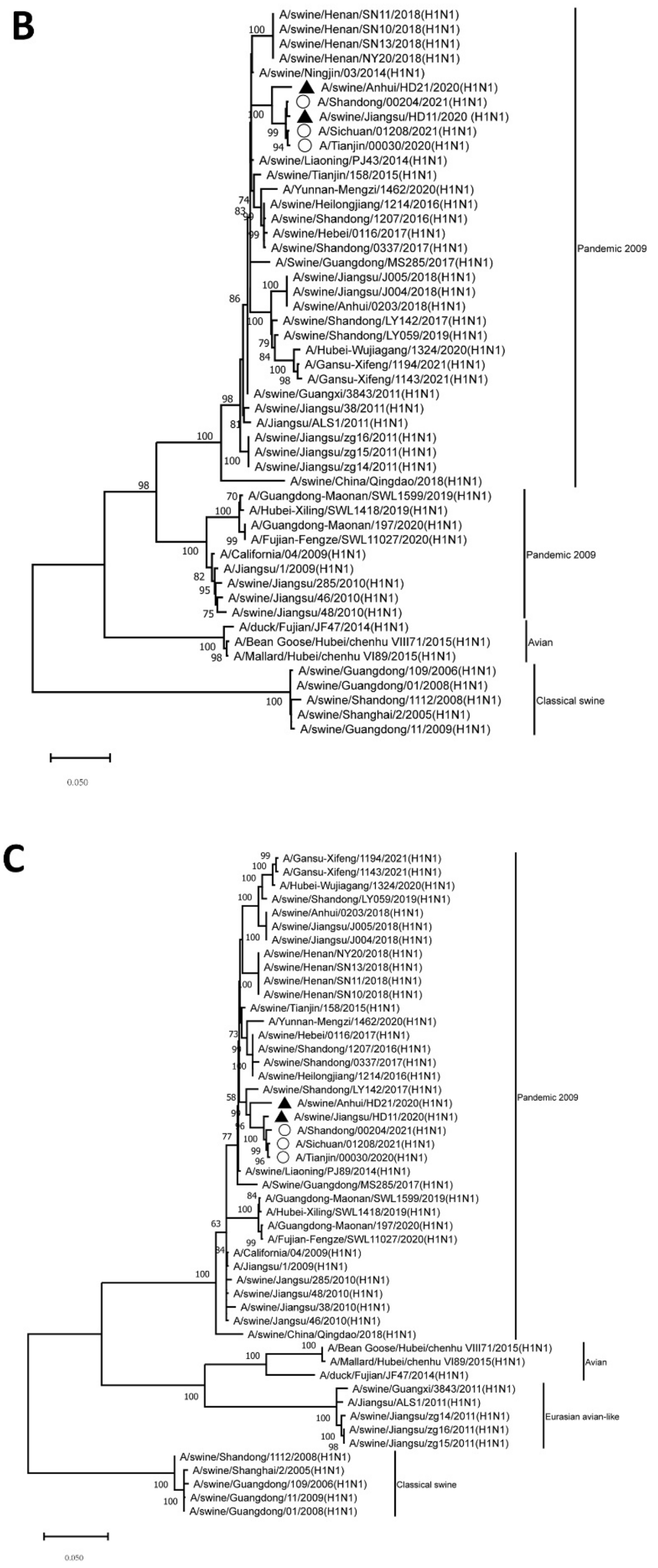
B
A/swine/Henan/SN11/2018(H1N1)
A/swine/Henan/SN10/2018(H1N1)
A/swine/Henan/SN13/2018(H1N1)
A/swine/Henan/NY20/2018(H1N1)
A/swine/Ningjin/03/2014(H1N1)
A/swine/Anhui/HD21/2020(H1N1)
A/Shandong/00204/2021(H1N1)
A/swine/Jiangsu/HD11/2020 (H1N1)
A/Sichuan/01208/2021(H1N1)
A/Tianjin/00030/2020(H1N1)
A/swine/Liaoning/PJ43/2014(H1N1)
A/swine/Tianjin/158/2015(H1N1)
A/Yunnan-Mengzi/1462/2020(H1N1)
A/swine/Heilongjiang/1214/2016(H1N1)
A/swine/Shandong/1207/2016(H1N1)
A/swine/Hebei/0116/2017(H1N1)
A/swine/Shandong/0337/2017(H1N1)
A/Swine/Guangdong/MS285/2017(H1N1)
A/swine/Jiangsu/J005/2018(H1N1)
A/swine/Jiangsu/J004/2018(H1N1)
A/swine/Anhui/0203/2018(H1N1)
A/swine/Shandong/LY142/2017(H1N1)
A/swine/Shandong/LY059/2019(H1N1)
A/Hubei-Wujiagang/1324/2020(H1N1)
A/Gansu-Xifeng/1194/2021(H1N1)
A/Gansu-Xifeng/1143/2021(H1N1)
A/swine/Guangxi/3843/2011(H1N1)
A/swine/Jiangsu/38/2011(H1N1)
A/Jiangsu/ALS1/2011(H1N1)
A/swine/Jiangsu/zg16/2011(H1N1)
A/swine/Jiangsu/zg15/2011(H1N1)
A/swine/Jiangsu/zg14/2011(H1N1)
A/swine/China/Qingdao/2018(H1N1)
Pandemic 2009
A/Guangdong-Maonan/SWL1599/2019(H1N1)
A/Hubei-Xiling/SWL1418/2019(H1N1)
A/Guangdong-Maonan/197/2020(H1N1)
A/Fujian-Fengze/SWL11027/2020(H1N1)
A/California/04/2009(H1N1)
A/Jiangsu/1/2009(H1N1)
A/swine/Jiangsu/285/2010(H1N1)
A/swine/Jiangsu/46/2010(H1N1)
A/swine/Jiangsu/48/2010(H1N1)
Pandemic 2009
A/duck/Fujian/JF47/2014(H1N1)
A/Bean Goose/Hubei/chenhu VIII71/2015(H1N1)
A/Mallard/Hubei/chenhu VI89/2015(H1N1)
Avian
A/swine/Guangdong/109/2006(H1N1)
A/swine/Guangdong/01/2008(H1N1)
A/swine/Shandong/1112/2008(H1N1)
A/swine/Shanghai/2/2005(H1N1)
A/swine/Guangdong/11/2009(H1N1)
Classical swine
0.050
C
A/Gansu-Xifeng/1194/2021(H1N1)
A/Gansu-Xifeng/1143/2021(H1N1)
A/Hubei-Wujiagang/1324/2020(H1N1)
A/swine/Shandong/LY059/2019(H1N1)
A/swine/Anhui/0203/2018(H1N1)
A/swine/Jiangsu/J005/2018(H1N1)
A/swine/Jiangsu/J004/2018(H1N1)
A/swine/Henan/NY20/2018(H1N1)
A/swine/Henan/SN13/2018(H1N1)
A/swine/Henan/SN11/2018(H1N1)
A/swine/Henan/SN10/2018(H1N1)
A/swine/Tianjin/158/2015(H1N1)
A/Yunnan-Mengzi/1462/2020(H1N1)
A/swine/Hebei/0116/2017(H1N1)
A/swine/Shandong/1207/2016(H1N1)
A/swine/Shandong/0337/2017(H1N1)
A/swine/Heilongjiang/1214/2016(H1N1)
A/swine/Shandong/LY142/2017(H1N1)
A/swine/Anhui/HD21/2020(H1N1)
A/swine/Jiangsu/HD11/2020(H1N1)
A/Shandong/00204/2021(H1N1)
A/Sichuan/01208/2021(H1N1)
A/Tianjin/00030/2020(H1N1)
A/swine/Liaoning/PJ89/2014(H1N1)
A/Swine/Guangdong/MS285/2017(H1N1)
A/Guangdong-Maonan/SWL1599/2019(H1N1)
A/Hubei-Xiling/SWL1418/2019(H1N1)
A/Guangdong-Maonan/197/2020(H1N1)
A/Fujian-Fengze/SWL11027/2020(H1N1)
A/California/04/2009(H1N1)
A/Jiangsu/1/2009(H1N1)
A/swine/Jangsu/285/2010(H1N1)
A/swine/Jiangsu/48/2010(H1N1)
A/swine/Jiangsu/38/2010(H1N1)
A/swine/Jangsu/46/2010(H1N1)
A/swine/China/Qingdao/2018(H1N1)
Pandemic 2009
A/Bean Goose/Hubei/chenhu VIII71/2015(H1N1)
A/Mallard/Hubei/chenhu VI89/2015(H1N1)
A/duck/Fujian/JF47/2014(H1N1)
Avian
A/swine/Guangxi/3843/2011(H1N1)
A/Jiangsu/ALS1/2011(H1N1)
A/swine/Jiangsu/zg14/2011(H1N1)
A/swine/Jiangsu/zg16/2011(H1N1)
A/swine/Jiangsu/zg15/2011(H1N1)
Eurasian avian-like
A/swine/Shandong/1112/2008(H1N1)
A/swine/Shanghai/2/2005(H1N1)
A/swine/Guangdong/109/2006(H1N1)
A/swine/Guangdong/11/2009(H1N1)
A/swine/Guangdong/01/2008(H1N1)
Classical swine
0.050

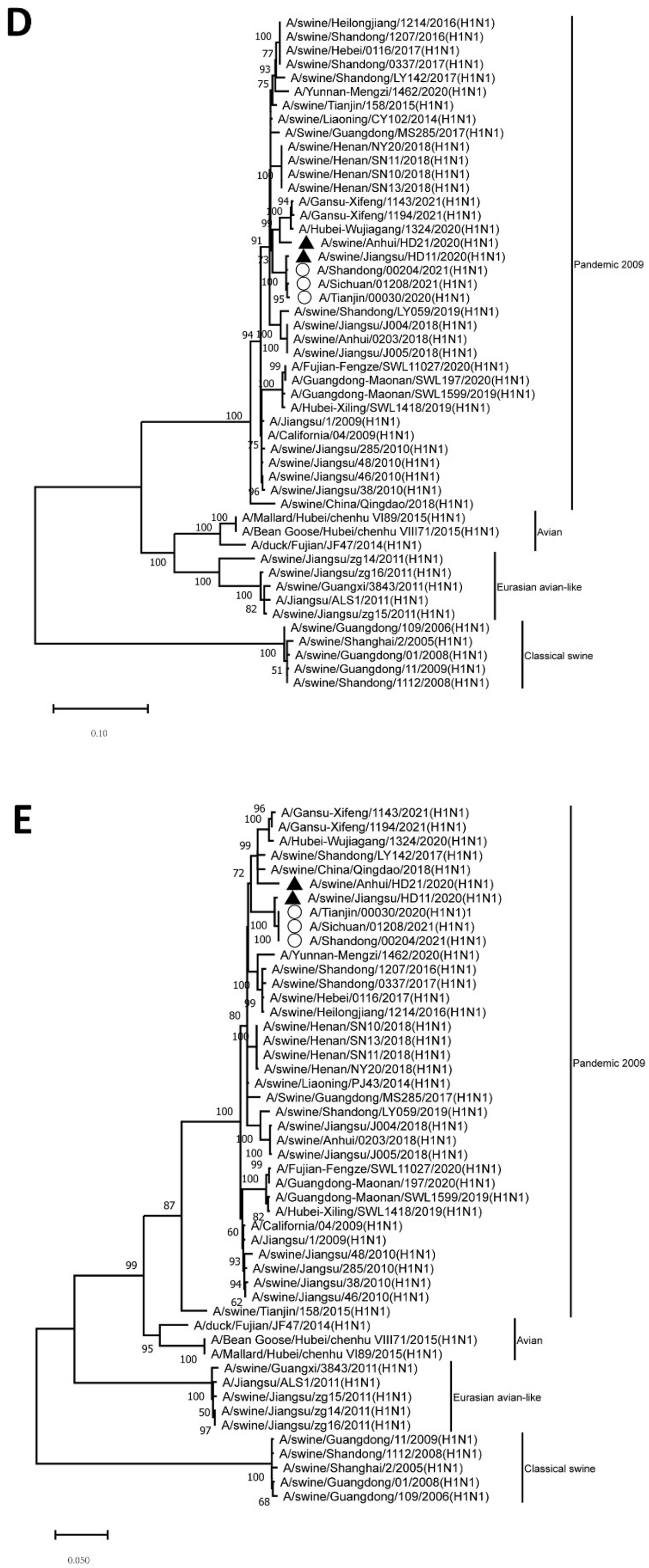
D
A/swine/Heilongjiang/1214/2016(H1N1)
A/swine/Shandong/1207/2016(H1N1)
A/swine/Hebei/0116/2017(H1N1)
A/swine/Shandong/0337/2017(H1N1)
A/swine/Shandong/LY142/2017(H1N1)
A/Yunnan-Mengzi/1462/2020(H1N1)
A/swine/Tianjin/158/2015(H1N1)
A/swine/Liaoning/CY102/2014(H1N1)
A/Swine/Guangdong/MS285/2017(H1N1)
A/swine/Henan/NY20/2018(H1N1)
A/swine/Henan/SN11/2018(H1N1)
A/swine/Henan/SN10/2018(H1N1)
A/swine/Henan/SN13/2018(H1N1)
A/Gansu-Xifeng/1143/2021(H1N1)
A/Gansu-Xifeng/1194/2021(H1N1)
A/Hubei-Wujiagang/1324/2020(H1N1)
A/swine/Anhui/HD21/2020(H1N1)
A/swine/Jiangsu/HD11/2020(H1N1)
A/Shandong/00204/2021(H1N1)
A/Sichuan/01208/2021(H1N1)
A/Tianjin/00030/2020(H1N1)
A/swine/Shandong/LY059/2019(H1N1)
A/swine/Jiangsu/J004/2018(H1N1)
A/swine/Anhui/0203/2018(H1N1)
A/swine/Jiangsu/J005/2018(H1N1)
A/Fujian-Fengze/SWL11027/2020(H1N1)
A/Guangdong-Maonan/SWL197/2020(H1N1)
A/Guangdong-Maonan/SWL1599/2019(H1N1)
A/Hubei-Xiling/SWL1418/2019(H1N1)
A/Jiangsu/1/2009(H1N1)
A/California/04/2009(H1N1)
A/swine/Jiangsu/285/2010(H1N1)
A/swine/Jiangsu/48/2010(H1N1)
A/swine/Jiangsu/46/2010(H1N1)
A/swine/Jiangsu/38/2010(H1N1)
A/swine/China/Qingdao/2018(H1N1)
A/Mallard/Hubei/chenhu VI89/2015(H1N1)
A/Bean Goose/Hubei/chenhu VIII71/2015(H1N1)
A/duck/Fujian/JF47/2014(H1N1)
A/swine/Jiangsu/zg14/2011(H1N1)
A/swine/Jiangsu/zg16/2011(H1N1)
A/swine/Guangxi/3843/2011(H1N1)
A/Jiangsu/ALS1/2011(H1N1)
A/swine/Jiangsu/zg15/2011(H1N1)
A/swine/Guangdong/109/2006(H1N1)
A/swine/Shanghai/2/2005(H1N1)
A/swine/Guangdong/01/2008(H1N1)
A/swine/Guangdong/11/2009(H1N1)
A/swine/Shandong/1112/2008(H1N1)
Pandemic 2009
Avian
Eurasian avian-like
Classical swine
0.10
E
A/Gansu-Xifeng/1143/2021(H1N1)
A/Gansu-Xifeng/1194/2021(H1N1)
A/Hubei-Wujiagang/1324/2020(H1N1)
A/swine/Shandong/LY142/2017(H1N1)
A/swine/China/Qingdao/2018(H1N1)
A/swine/Anhui/HD21/2020(H1N1)
A/swine/Jiangsu/HD11/2020(H1N1)
A/Tianjin/00030/2020(H1N1)1
A/Sichuan/01208/2021(H1N1)
A/Shandong/00204/2021(H1N1)
A/Yunnan-Mengzi/1462/2020(H1N1)
A/swine/Shandong/1207/2016(H1N1)
A/swine/Shandong/0337/2017(H1N1)
A/swine/Hebei/0116/2017(H1N1)
A/swine/Heilongjiang/1214/2016(H1N1)
A/swine/Henan/SN10/2018(H1N1)
A/swine/Henan/SN13/2018(H1N1)
A/swine/Henan/SN11/2018(H1N1)
A/swine/Henan/NY20/2018(H1N1)
A/swine/Liaoning/PJ43/2014(H1N1)
A/Swine/Guangdong/MS285/2017(H1N1)
A/swine/Shandong/LY059/2019(H1N1)
A/swine/Jiangsu/J004/2018(H1N1)
A/swine/Anhui/0203/2018(H1N1)
A/swine/Jiangsu/J005/2018(H1N1)
A/Fujian-Fengze/SWL11027/2020(H1N1)
A/Guangdong-Maonan/197/2020(H1N1)
A/Guangdong-Maonan/SWL1599/2019(H1N1)
A/Hubei-Xiling/SWL1418/2019(H1N1)
A/California/04/2009(H1N1)
A/Jiangsu/1/2009(H1N1)
A/swine/Jiangsu/48/2010(H1N1)
A/swine/Jangsu/285/2010(H1N1)
A/swine/Jiangsu/38/2010(H1N1)
A/swine/Jiangsu/46/2010(H1N1)
A/swine/Tianjin/158/2015(H1N1)
A/duck/Fujian/JF47/2014(H1N1)
A/Bean Goose/Hubei/chenhu VIII71/2015(H1N1)
A/Mallard/Hubei/chenhu VI89/2015(H1N1)
A/swine/Guangxi/3843/2011(H1N1)
A/Jiangsu/ALS1/2011(H1N1)
A/swine/Jiangsu/zg15/2011(H1N1)
A/swine/Jiangsu/zg14/2011(H1N1)
A/swine/Jiangsu/zg16/2011(H1N1)
A/swine/Guangdong/11/2009(H1N1)
A/swine/Shandong/1112/2008(H1N1)
A/swine/Shanghai/2/2005(H1N1)
A/swine/Guangdong/01/2008(H1N1)
A/swine/Guangdong/109/2006(H1N1)
Pandemic 2009
Avian
Eurasian avian-like
Classical swine
0.050

F

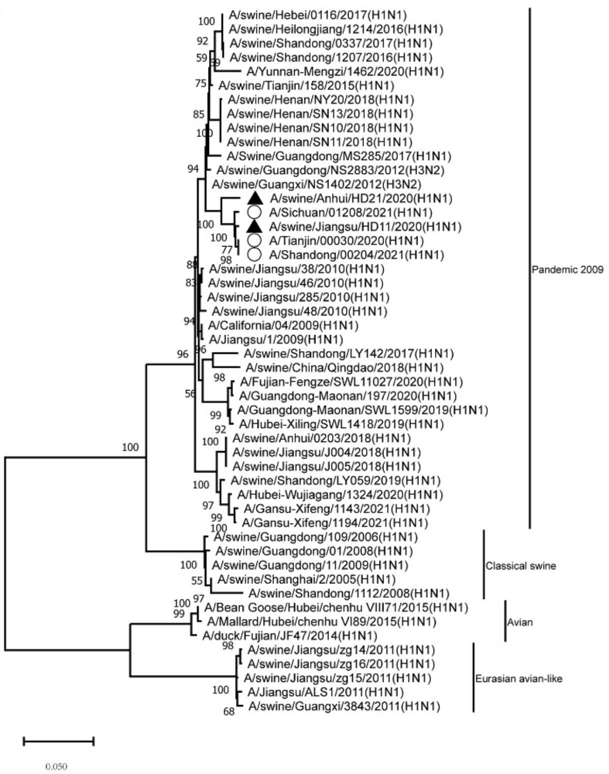
A/swine/Hebei/0116/2017(H1N1)
A/swine/Heilongjiang/1214/2016(H1N1)
A/swine/Shandong/0337/2017(H1N1)
A/swine/Shandong/1207/2016(H1N1)
A/Yunnan-Mengzi/1462/2020(H1N1)
A/swine/Tianjin/158/2015(H1N1)
A/swine/Henan/NY20/2018(H1N1)
A/swine/Henan/SN13/2018(H1N1)
A/swine/Henan/SN10/2018(H1N1)
A/swine/Henan/SN11/2018(H1N1)
A/Swine/Guangdong/MS285/2017(H1N1)
A/swine/Guangdong/NS2883/2012(H3N2)
A/swine/Guangxi/NS1402/2012(H3N2)
A/swine/Anhui/HD21/2020(H1N1)
A/Sichuan/01208/2021(H1N1)
A/swine/Jiangsu/HD11/2020(H1N1)
A/Tianjin/00030/2020(H1N1)
A/Shandong/00204/2021(H1N1)
A/swine/Jiangsu/38/2010(H1N1)
A/swine/Jiangsu/46/2010(H1N1)
A/swine/Jiangsu/285/2010(H1N1)
A/swine/Jiangsu/48/2010(H1N1)
A/California/04/2009(H1N1)
A/Jiangsu/1/2009(H1N1)
A/swine/Shandong/LY142/2017(H1N1)
A/swine/China/Qingdao/2018(H1N1)
A/Fujian-Fengze/SWL11027/2020(H1N1)
A/Guangdong-Maonan/197/2020(H1N1)
A/Guangdong-Maonan/SWL1599/2019(H1N1)
A/Hubei-Xiling/SWL1418/2019(H1N1)
A/swine/Anhui/0203/2018(H1N1)
A/swine/Jiangsu/J004/2018(H1N1)
A/swine/Jiangsu/J005/2018(H1N1)
A/swine/Shandong/LY059/2019(H1N1)
A/Hubei-Wujiagang/1324/2020(H1N1)
A/Gansu-Xifeng/1143/2021(H1N1)
A/Gansu-Xifeng/1194/2021(H1N1)
A/swine/Guangdong/109/2006(H1N1)
A/swine/Guangdong/01/2008(H1N1)
A/swine/Guangdong/11/2009(H1N1)
A/swine/Shanghai/2/2005(H1N1)
A/swine/Shandong/1112/2008(H1N1)
A/Bean Goose/Hubei/chenhu VIII71/2015(H1N1)
A/Mallard/Hubei/chenhu VI89/2015(H1N1)
A/duck/Fujian/JF47/2014(H1N1)
A/swine/Jiangsu/zg14/2011(H1N1)
A/swine/Jiangsu/zg16/2011(H1N1)
A/swine/Jiangsu/zg15/2011(H1N1)
A/Jiangsu/ALS1/2011(H1N1)
A/swine/Guangxi/3843/2011(H1N1)
Pandemic 2009
Classical swine
Avian
Eurasian avian-like
0.050

G

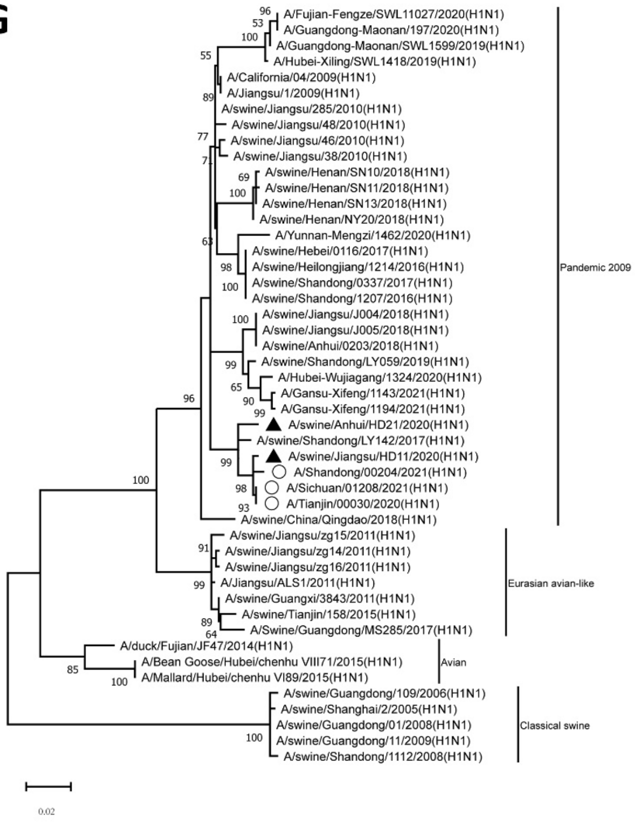
A/Fujian-Fengze/SWL11027/2020(H1N1)
A/Guangdong-Maonan/197/2020(H1N1)
A/Guangdong-Maonan/SWL1599/2019(H1N1)
A/Hubei-Xiling/SWL1418/2019(H1N1)
A/California/04/2009(H1N1)
A/Jiangsu/1/2009(H1N1)
A/swine/Jiangsu/285/2010(H1N1)
A/swine/Jiangsu/48/2010(H1N1)
A/swine/Jiangsu/46/2010(H1N1)
A/swine/Jiangsu/38/2010(H1N1)
A/swine/Henan/SN10/2018(H1N1)
A/swine/Henan/SN11/2018(H1N1)
A/swine/Henan/SN13/2018(H1N1)
A/swine/Henan/NY20/2018(H1N1)
A/Yunnan-Mengzi/1462/2020(H1N1)
A/swine/Hebei/0116/2017(H1N1)
A/swine/Heilongjiang/1214/2016(H1N1)
A/swine/Shandong/0337/2017(H1N1)
A/swine/Shandong/1207/2016(H1N1)
A/swine/Jiangsu/J004/2018(H1N1)
A/swine/Jiangsu/J005/2018(H1N1)
A/swine/Anhui/0203/2018(H1N1)
A/swine/Shandong/LY059/2019(H1N1)
A/Hubei-Wujiagang/1324/2020(H1N1)
A/Gansu-Xifeng/1143/2021(H1N1)
A/Gansu-Xifeng/1194/2021(H1N1)
A/swine/Anhui/HD21/2020(H1N1)
A/swine/Shandong/LY142/2017(H1N1)
A/swine/Jiangsu/HD11/2020(H1N1)
A/Shandong/00204/2021(H1N1)
A/Sichuan/01208/2021(H1N1)
A/Tianjin/00030/2020(H1N1)
A/swine/China/Qingdao/2018(H1N1)
A/swine/Jiangsu/zg15/2011(H1N1)
A/swine/Jiangsu/zg14/2011(H1N1)
A/swine/Jiangsu/zg16/2011(H1N1)
A/Jiangsu/ALS1/2011(H1N1)
A/swine/Guangxi/3843/2011(H1N1)
A/swine/Tianjin/158/2015(H1N1)
A/Swine/Guangdong/MS285/2017(H1N1)
A/duck/Fujian/JF47/2014(H1N1)
A/Bean Goose/Hubei/chenhu VIII71/2015(H1N1)
A/Mallard/Hubei/chenhu VI89/2015(H1N1)
A/swine/Guangdong/109/2006(H1N1)
A/swine/Shanghai/2/2005(H1N1)
A/swine/Guangdong/01/2008(H1N1)
A/swine/Guangdong/11/2009(H1N1)
A/swine/Shandong/1112/2008(H1N1)
Pandemic 2009
Eurasian avian-like
Avian
Classical swine
0.02

H

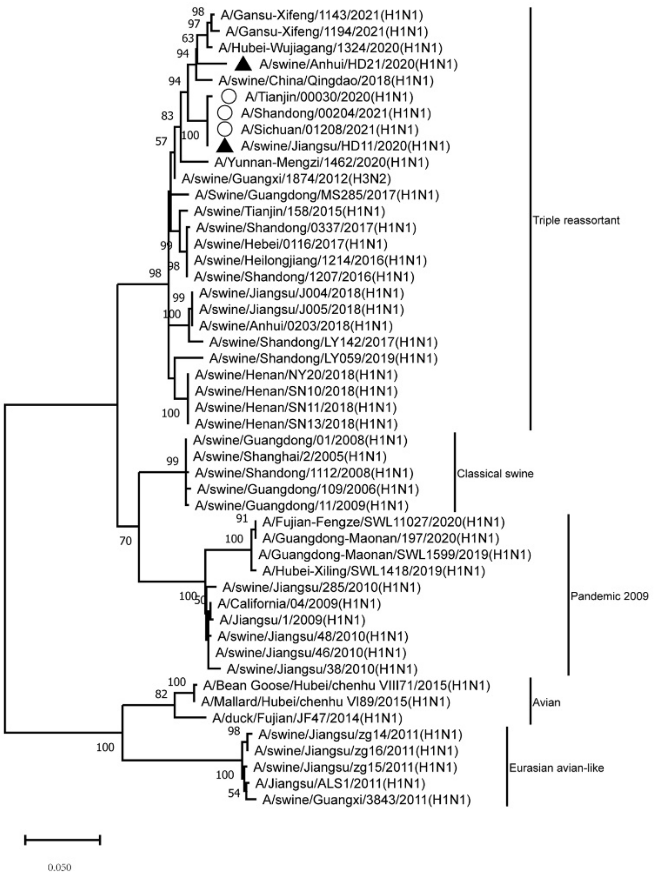

Black triangles indicate G4 EA H1N1 swine isolates of A/swine/Jiangsu/HD11/2020(H1N1) [HD11] and A/swine/Anhui/HD21/2020(H1N1) [HD21] in this study. Reference sequences of Eurasian avian-like, Pandemic 2009, Classical swine, Triple reassortant and avian H1N1 subtype IAV lineages were retrieved from the GISAID platform (http://www.gisaid.org), especially those human viruses showing consistent homology with HD11 and HD21 in all 8 genes; hollow circles indicate those strains. Each maximum-likelihood tree was generated using the MEGA software version 11 (http://www.megasoftware.net). We evaluated tree topology by bootstrap resampling method with 1,000 replicates. Scale bar indicates nucleotide substitutions per site.

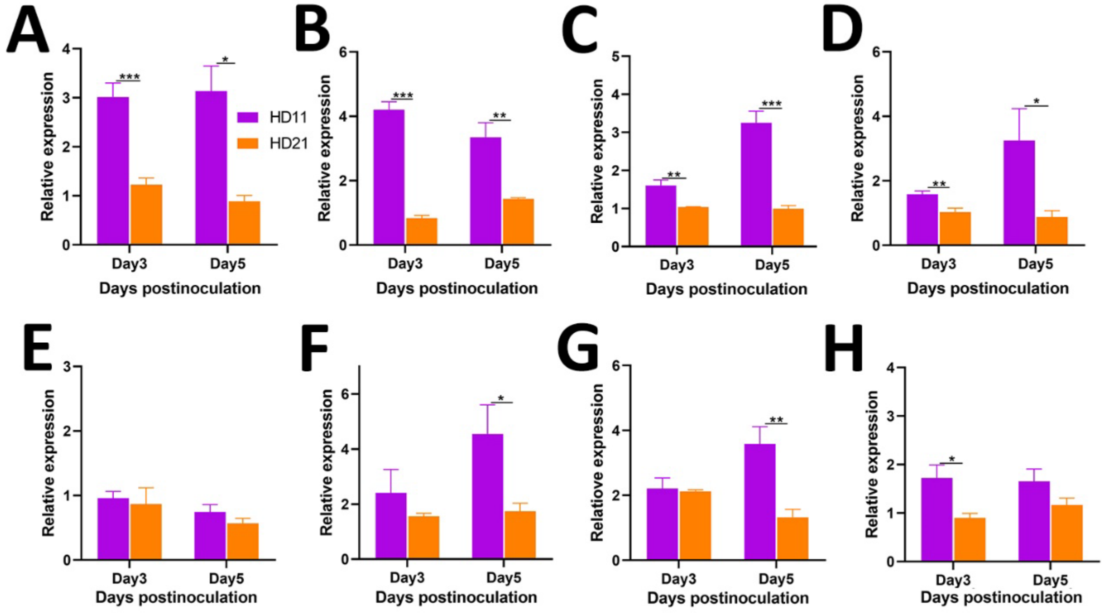

Appendix Figure 2. The expression level of inflammatory cytokines induced by 2 G4 Eurasian avian-like H1N1 swine isolates in mouse lungs. Groups of six 6-week-old BABL/c mice were intranasally inoculated with $10^{6.0}$ EID_{50} A/swine/Jiangsu/HD11/2020(H1N1) [HD11] or A/swine/Anhui/HD21/2020(H1N1) [HD21]. On 3 and 5 days postinoculation, 3 mice from each group were euthanized to collect the lungs for homogenate preparation. We evaluated relative mRNA expression levels of IL-6 (A), IL-10 (B), IFN-β (C), INF-γ (D), TNF-α (E), MX1 (F), CXCL-10 (G), and CXCL-11 (H) in the infected mouse lungs by the real-time quantitative RT-PCR method. All values were normalized to GAPDH and expressed as fold change against controls. Values were shown as mean±SD from 3 independent determinations. (*, $p<0.05$; **, $p<0.01$; ***, $p<0.001$).
